# Radiographic Analysis of Early Changes in Upper Adjacent Segments After Fusion Surgery: OLIF vs. PLIF

**DOI:** 10.3390/jcm14186570

**Published:** 2025-09-18

**Authors:** JooYoung Lee, JaeHwan Cho, Dong-Ho Lee, ChangJu Hwang, SeHan Park

**Affiliations:** 1 Department of Orthopedic Surgery, Ulsan University Hospital, University of Ulsan College of Medicine, Ulsan 44033, Republic of Korea; forsky07@naver.com; 2 Department of Orthopedic Surgery, Asan Medical Center, University of Ulsan College of Medicine, Seoul 05505, Republic of Korea; osdlee@gmail.com (D.-H.L.); baski47@gmail.com (C.H.); birdone86@gmail.com (S.P.)

**Keywords:** OLIF, PLIF, adjacent segment degeneration, radiographic changes, lumbar fusion

## Abstract

**Background:** Oblique lumbar interbody fusion (OLIF) has recently gained popularity as a minimally invasive surgical technique for lumbar fusion. While OLIF is superior in restoring disc height and lumbar lordosis compared to posterior lumbar interbody fusion (PLIF), its biomechanical effect on adjacent segments remains unclear. **Methods:** We retrospectively analyzed 236 patients who underwent one- or two-level OLIF (*n* = 95) or PLIF (*n* = 141) between 2013 and 2020. Radiographic outcomes, including lumbar lordosis, upper adjacent segmental lordosis, retrolisthesis, and foraminal height, were evaluated preoperatively and at 3 days and 1, 3, 6, and 12 months postoperatively. Patient-reported outcomes (VAS for back/leg pain and Oswestry Disability Index [ODI]) were assessed preoperatively and at 12 months. **Results:** OLIF provided superior restoration of lumbar lordosis (4.03 ± 4.38° vs. 1.63 ± 5.11°, *p* = 0.001) and disc height (5.50 ± 3.39 mm vs. 2.71 ± 2.18 mm, *p* < 0.0001) compared with PLIF. However, OLIF was associated with higher incidence (76.9% vs. 24.6%, *p* < 0.0001) and degree of retrolisthesis (1.69 ± 1.09 mm vs. 0.29 ± 0.70 mm, *p* < 0.0001), and decreased foraminal height (−1.43 ± 2.12 mm vs. 0.54 ± 2.53 mm, *p* < 0.0001) in the upper adjacent segment. Importantly, there was no significant difference in clinical outcomes (VAS and ODI) between the two groups at 12 months (all *p* > 0.05). **Conclusions:** While OLIF achieves superior restoration of lumbar lordosis and disc height compared to PLIF, it also induces early radiographic deterioration in the upper adjacent segment. Importantly, these findings represent radiographic changes observed within 1 year, without significant differences in clinical outcomes, and longer-term follow-up is required to determine their clinical relevance.

## 1. Introduction

Spinal fusion surgery is a widely employed technique for treating various spinal pathologies, such as degenerative disc disease, spinal stenosis, and spondylolisthesis [1]. Despite its effectiveness in achieving spinal stability and alleviating symptoms, fusion surgery often alters the biomechanics of the spine, potentially leading to adjacent segment degeneration (ASD) in the levels adjacent to the fused segment [2]. ASD is a well-recognized complication characterized by progressive degeneration of the spinal segments adjacent to the fusion, resulting in clinical symptoms and necessitating additional surgical interventions in some cases [3]. Among the techniques utilized in spinal fusion surgery, two common approaches include Oblique Lumbar Interbody Fusion (OLIF) and Posterior Lumbar Interbody Fusion (PLIF). OLIF is a minimally invasive procedure that allows access to the intervertebral disc space through a lateral approach, avoiding significant disruption to the posterior spinal elements. In contrast, PLIF involves a posterior midline incision with direct access to the intervertebral disc space, often necessitating extensive resection of the posterior elements for adequate decompression and fusion [4].

Previous studies have reported that there are minimal differences among surgical techniques using the posterior approach [5,6]. However, the differences between these techniques and OLIF have not yet been clearly established [7,8]. In particular, studies on the early effects of the two surgical techniques on adjacent segments are extremely limited [9]. While both OLIF and PLIF are effective in achieving fusion and decompression, their impact on the development of ASD remains a subject of debate. Previous studies have investigated the incidence and risk factors associated with ASD following these surgical techniques [10], but there is a paucity of literature comparing radiographic changes in the upper adjacent segments between OLIF and PLIF.

Understanding the early radiographic changes in the upper adjacent segments following fusion surgery is crucial for optimizing surgical strategies and minimizing the risk of ASD. Therefore, this study aims to conduct a comparative analysis of radiographic changes in the upper adjacent segments after OLIF and PLIF procedures. By elucidating the differences in the biomechanical effects of these surgical techniques on adjacent segment dynamics, this study seeks to provide valuable insights into the long-term outcomes and complications associated with spinal fusion surgery. Ultimately, such findings may contribute to the refinement of surgical techniques and the development of preventive strategies to mitigate the risk of ASD and improve the overall success rate of spinal fusion procedures.

## 2. Materials and Methods

### 2.1. Patients

After obtaining Asan Medical Center Institutional Review Board approval (IRB#2023-0108; 11 October 2023), this study was conducted using the details of 236 consecutive patients who underwent 1 or 2 level OLIF or PLIF surgery by single surgeon between January 2013 and January 2020 at a single academic institution and completed a 1-year follow-up, comprising two groups: Group P (PLIF group, *n* = 141) and Group O (OLIF group, *n* = 95). As this study did not involve identifiable patient data and was conducted retrospectively with IRB approval, the requirement for informed consent was waived. The study utilized a matched pairs design, ensuring comparability between the two groups based on relevant demographic and clinical characteristics. Patients with degenerative spondylolisthesis (Meyerding classification grade is I [11]) accompanied by Schizas classification grade C or D severe spinal stenosis or grade 3 neuroforaminal stenosis [12], who failed conservative treatment for more than three months, were considered candidates for surgical treatment. Underwent comprehensive radiographic evaluations, including preoperative, intraoperative, and postoperative assessments at specified time points. Provided complete data on patient-reported outcome measures (PROMs) collected both preoperatively and at a 1-year follow-up. Patients meeting any of the following criteria were excluded from the study. History of previous lumbar spine surgery. Presence of congenital spinal abnormalities or deformities. Diagnosis of inflammatory or infective spinal pathologies. Incomplete data on radiographic evaluations or PROMs. Presence of significant comorbidities that could potentially confound the study outcomes. Baseline demographic and clinical characteristics of the patients in both groups were recorded and compared. These characteristics included age, gender, body mass index (BMI), smoking status, comorbidities (such as diabetes mellitus, hypertension), preoperative diagnosis level, and severity of symptoms assessed using standardized clinical evaluation tools.

### 2.2. Radiographic Evaluation

**Figure 1:**
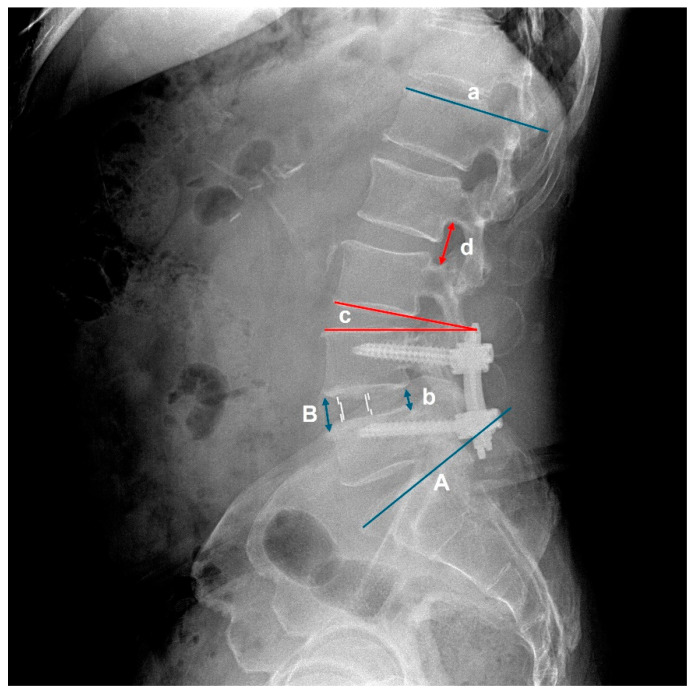
Image demonstrating the measurement of lumbar lordosis (A~a), disc height ((B + b)/2), segmental lordosis (c), and foraminal height (d).

All patients underwent standardized upright long-cassette lateral radiography in a neutral standing position with arms folded across the chest and eyes facing forward. Images were obtained during a breath-hold to minimize motion artifacts, and a calibration marker was included in each image to ensure measurement accuracy. Each patient underwent plain upright whole spine lateral radiography preoperatively, as well as at 3 days, 1 month, 3 months, 6 months, and 1 year postoperatively. Radiographic parameters including lumbar lordosis, upper adjacent segmental lordosis, retrolisthesis, and foraminal height were measured at each time point. Changes in these radiographic values from the preoperative baseline were recorded and analyzed to assess early changes in the upper adjacent segments following fusion surgery. To compare the degree of degeneration of adjacent segments before surgery, disc degeneration (Pfirrmann Grading system [13]) was measured using MRI images, and facet sagitallization (°) and facet degeneration (Weishaupt Grading system [14]) were measured and compared using CT images. Among the co-authors, two orthopedic surgeons specializing in the lumbar spine directly analyzed the blinded plain radiography, CT, and MRI images of 241 patients. All images are de-identified, arranged in random order and distributed. Each image included a uniform calibration scale. Lumbar lordosis was defined as the angle between lines parallel to the upper endplates of L1 and S1. Segmental lordosis was defined as the angle between lines parallel to the lower endplate of the superior vertebral body and the upper endplate of the inferior vertebral body at the corresponding level. Disc height was measured as the average length of lines connecting the anterior and posterior margins of the lower endplate of the superior vertebral body and the upper endplate of the inferior vertebral body at the respective disc level. Flexibility was defined as the range of motion (ROM) measured on dynamic flexion–extension lateral radiographs. Retrolisthesis was defined as posterior displacement of more than 2 mm on lateral radiographs, measured as the distance between the posterior vertebral body lines of the adjacent segments. The incidence of retrolisthesis was defined by drawing parallel lines along the posterior margins of each vertebral body, and if any degree of posterior displacement was observed, it was considered positive and calculated as a percentage (Figure 1). Measurements were performed in the order listed, and Petavision 2.0 (Asan medical center, Seoul, Republic of Korea) software was used. Each orthopedic rater was trained on the same measurement method and repeated the measurement three times. To evaluate measurement reliability, intra- and inter-rater intraclass correlation coefficients (ICCs) were calculated for all radiographic variables (lumbar lordosis, segmental lordosis, disc height, foraminal height, and retrolisthesis). The ICCs ranged from 0.82 to 0.94, indicating good to excellent reliability.

### 2.3. Patient-Reported Outcome Measures

Preoperatively and at 1-year follow-up, patient-reported outcome measures were obtained to assess the clinical effectiveness and patient satisfaction following surgery. Standardized PROMs tools, such as the Visual Analogue Scale (VAS) for back and leg pain, and Oswestry Disability Index (ODI) for functional disability were administered to all patients.

### 2.4. Surgical Methods

OLIF is a minimally invasive surgical technique utilized for achieving lumbar spinal fusion and decompression. The procedure involves accessing the intervertebral disc space through a lateral approach, typically performed through the patient’s flank. Key steps in the OLIF procedure include: The patient is placed in a lateral decubitus position on the operating table, with the left side facing upward. Proper padding and positioning aids in optimizing access to the target intervertebral disc space. A small incision is made on the flank of the patient, typically at the level of the targeted intervertebral disc. Through this incision, the surgeon gains access to the retroperitoneal space, allowing for direct visualization of the lumbar spine without significant disruption to the surrounding muscles and tissues. After accessing the target disc space, a discectomy is performed to remove the degenerated or herniated disc material. Specialized instruments are used to prepare the endplates of the adjacent vertebral bodies, creating a suitable environment for fusion. A specially designed interbody cage, typically made of biocompatible materials such as polyetheretherketone (PEEK), is inserted into the disc space. The cage serves to restore disc height, correct spinal alignment, and facilitate fusion between the adjacent vertebral bodies. After suturing layer by layer, change the patient’s position to prone and perform percutaneous pedicle screw fixation under C-arm guidance.

Posterior Lumbar Interbody Fusion (PLIF) is a conventional surgical technique employed for achieving lumbar spinal fusion and decompression. Unlike OLIF, PLIF involves a posterior midline incision and direct access to the intervertebral disc space through the posterior approach. Key steps in the PLIF procedure include: The patient is positioned prone on the operating table, with proper padding and positioning to optimize exposure of the lumbar spine. A midline incision is made in the lower back, extending from the spinous processes of the involved vertebrae. The muscles and soft tissues are dissected to expose the laminae and facet joints of the targeted lumbar segments. Insert each pedicle screw by targeting the intersection of the line bisecting the transversal process and the outer surface of the superior articular process. To gain access to the intervertebral disc space, a partial or complete laminectomy and facetectomy may be performed. This involves removal of the lamina and portions of the facet joints to create sufficient space for instrumentation and fusion. The degenerated or herniated disc material is meticulously removed, and the endplates of the adjacent vertebral bodies are prepared using specialized instruments to facilitate fusion. Similarly to OLIF, an interbody cage is inserted into the disc space to restore disc height, correct spinal alignment, and promote fusion between the adjacent vertebral bodies. Bone graft material is packed into the interbody space to promote fusion. Pedicle screws are typically employed for posterior fixation, providing stability and support to the treated segment during the fusion process.

The selection of surgical approach (OLIF vs. PLIF) during the study period was influenced primarily by the evolving institutional practice patterns and growing clinical experience rather than strict predefined criteria. In the earlier years of the study (2013–2016), PLIF was more commonly employed due to its established surgical familiarity and outcomes. As clinical experience with OLIF accumulated and favourable short-term outcomes were observed, there was a gradual shift in preference toward OLIF in the latter years (2017–2020). Thus, the choice of surgical technique in this cohort reflects a natural progression of surgical strategy based on accumulating evidence, surgeon familiarity, and evolving institutional protocols, rather than patient-specific anatomical or clinical selection criteria.

### 2.5. Statistical Methods

Differences in proportions between the groups were analyzed using the chi-square test. Continuous variables were compared using both parametric (Student’s *t*-test) and non-parametric tests (Mann–Whitney U test for independent samples and Wilcoxon’s signed-rank test for dependent samples), as appropriate. Normality of continuous variables was assessed using the Shapiro–Wilk test prior to applying parametric or non-parametric statistical analyses.

Statistical analysis was performed using SPSS version 20.0 (SPSS Inc., Chicago, IL, USA) and MedCalc version 20.106 (MedCalc Software Ltd., Acacialaan, Ostend, Belgium). Statistical significance was set at *p* < 0.05.

## 3. Results

The mean age in Group O was 65.07 years (±8.22), slightly higher than in Group P, which was 63.41 years (±7.68). However, this difference was not statistically significant (*p* = 0.133). There were no statistical differences between the two groups in terms of gender, smoking status, surgical level, BMI, and comorbidities. There were no significant differences in lumbar lordosis and pelvic incidence between the two groups preoperatively (*p* = 0.502 and *p* = 0.216, respectively). Mean preoperative disc height was similar between Group O (7.80 ± 3.16 mm) and Group P (7.91 ± 2.44 mm), with no statistically significant difference observed (*p* = 0.487). Preoperative upper adjacent segmental degenerative radiologic parameters, such as foraminal height (group O: 19.53 ± 2.05, group P: 20.1 ± 3.01, *p* = 0.194), segmental lordosis (group O: 8.15 ± 3.18, group P: 7.75 ± 3.88, *p* = 0.379), flexibility in dynamic lumbar spine lateral radiography (group O: 7.64 ± 2.05, group P: 7.49 ± 3.88, *p* = 0.515), disc degeneration (Pfirrmann Grade, group O: 2.33 ± 0.062, group P: 2.44 ± 0.59, *p* = 0.267), facet sagittalization (°) (group O: 69.16 ± 9.04, group P: 71.19 ± 12.62, *p* = 0.259), and facet degeneration (Weishaupt Grade, group O: 2.16 ± 0.69, group P: 2.36 ± 0.48, *p* = 0.085), did not significantly differ between the two groups. Both groups showed similar preoperative outcomes in terms of Oswestry Disability Index (ODI, group O: 48.71 ± 8.23, group P: 45.39 ± 10.39, *p* = 0.81), back pain Visual Analogue Scale (VAS, group O: 5.47 ± 2.71, group P: 5.98 ± 2.69, *p* = 0.75), and leg pain VAS (group O: 8.51 ± 1.16, group P: 7.61 ± 1.94, *p* = 0.63), with no statistically significant differences observed (Table 1). In all 201 patients, there was no retrolisthesis of the upper adjacent segment at the planned surgical level prior to surgery.

**Figure 2:**
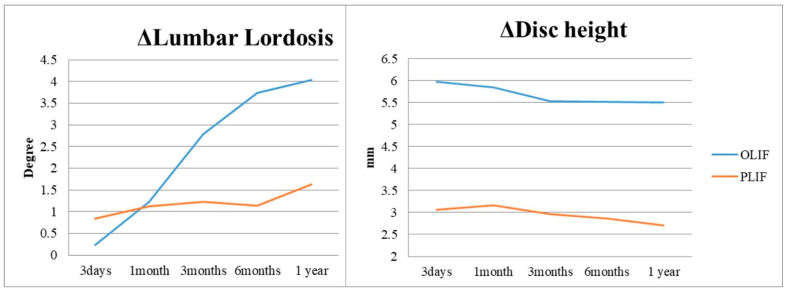
Changes in lumbar lordosis (degrees) and disc height (mm) at the surgical level over time (preoperative, postoperative day 3, 1 month, 3 months, 6 months, and 12 months) in the OLIF and PLIF groups.

**Figure 3:**
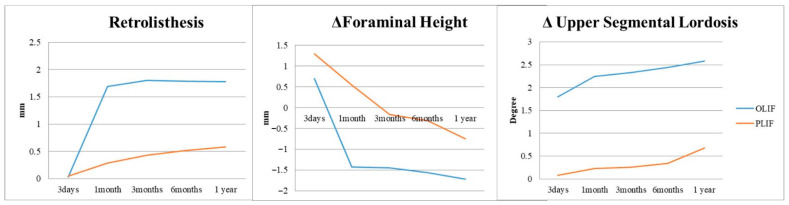
Changes in upper adjacent segment parameters over time in the OLIF and PLIF groups: retrolisthesis incidence (%), retrolisthesis degree (mm), foraminal height (mm), and segmental lordosis (degrees), measured at preoperative, postoperative day 3, 1 month, 3 months, 6 months, and 12 months.

**Figure 4:**
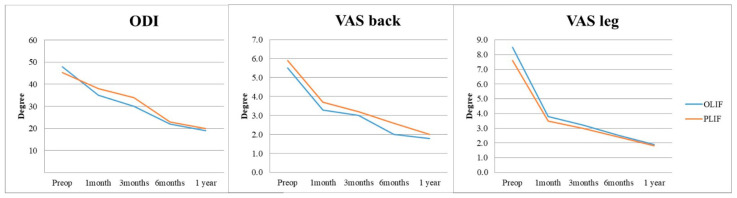
Changes in patient-reported outcomes in the OLIF and PLIF groups: Oswestry Disability Index (ODI, %), VAS for back pain (0–10), and VAS for leg pain (0–10), assessed at preoperative baseline and at 12-month follow-up.

In the 1-year postoperative period, Group O demonstrated superior increase in lumbar lordosis (4.03 ± 4.38°) compared to Group P (1.63 ± 5.11°), indicating OLIF’s effectiveness in this aspect (*p* = 0.001). Similarly, Group O exhibited a significant increase in surgical segmental disc height (5.50 ± 3.39 mm) compared to Group P (2.71 ± 2.18 mm) at the 1-year follow-up (Figure 2 and Table 2), highlighting OLIF’s advantage in disc height restoration (*p* < 0.001). At 3 days postoperatively, Group O showed a notable increase in upper adjacent segmental lordosis (1.8 ± 4.39°) compared to Group P (0.08 ± 3.35°), indicating an immediate effect of OLIF on the upper adjacent segment (*p* = 0.001). Furthermore, Group O exhibited a significantly higher incidence of retrolisthesis (76.9%) in the upper adjacent segment compared to Group P (24.6%) at 1 month postoperatively, along with a greater degree of retrolisthesis (1.69 ± 1.09 mm for Group O, 0.29 ± 0.70 mm for Group P) (*p* < 0.001). Additionally, Group O demonstrated a considerable decrease in foraminal height of the upper adjacent segment (−1.43 ± 2.12 mm) compared to Group P (0.54 ± 2.53 mm) at 1 month postoperatively, indicating a potential impact of OLIF on foraminal dimensions in the upper adjacent segment (*p* < 0.001) (Figure 3, Table 2). Although OLIF demonstrated superior restoration of lumbar lordosis and disc height, there were no significant differences between the two groups in patient-reported outcomes, including ODI, VAS for back pain, and VAS for leg pain, at 12 months postoperatively (all *p* > 0.05). There was no statistically significant difference between the two groups in terms of improvement in clinical outcomes, including ODI, VAS back, and VAS leg, after surgery (Figure 4). The intra- and inter-rater ICCs for radiographic measurements were consistently high (0.82–0.94), confirming good reproducibility.

## 4. Discussion

Oblique Lumbar Interbody Fusion (OLIF) and Posterior Lumbar Interbody Fusion (PLIF) are two prominent surgical techniques employed for the treatment of degenerative lumbar conditions [15,16]. OLIF is characterized by its minimally invasive approach, allowing access to the intervertebral disc space through a lateral incision. This technique minimizes disruption to the posterior spinal elements, potentially reducing postoperative pain and promoting quicker recovery [8,17,18]. In contrast, PLIF is a more traditional method involving a posterior midline incision, providing direct access to the disc space but often requiring extensive resection of surrounding structures for adequate decompression and stabilization [4,19].

Both OLIF and PLIF aim to achieve spinal fusion and relief symptoms associated with lumbar pathologies, but they differ significantly in their surgical approach, impact on surrounding tissues, and potential influences for adjacent segment dynamics [4,20]. While studies exist comparing the advantages and disadvantages of each surgical technique and their relationship with adjacent segment degeneration (ASD) [21,22], there has yet to be a comparative study specifically focusing on the adjacent segments between PLIF and OLIF. Some studies have reported that preoperative adjacent facet joint osteoarthritis may be associated with the development of radiological adjacent segment degeneration following spinal fusion surgery [23,24]. The findings of our study contribute to the ongoing discourse regarding the comparative effectiveness of OLIF and PLIF in lumbar spinal fusion surgery, particularly concerning their impact on adjacent segment dynamics and the risk of early ASD.

Consistent with previous research, our study demonstrates that OLIF exhibits superior capability over PLIF in restoring lumbar lordosis and surgical segmental disc height at the 1-year postoperative mark. The significant improvements observed in lumbar lordosis and disc height restoration following OLIF underscore its efficacy in achieving spinal realignment and addressing disc pathology, which are critical objectives in spinal fusion surgery. The minimally invasive nature of OLIF and the characteristic that allows for the insertion of a higher cage in the surgical technique of OLIF likely contribute to these favourable outcomes, as it allows for targeted disc decompression and restoration without the need for extensive disruption of posterior spinal elements.

**Figure 5:**
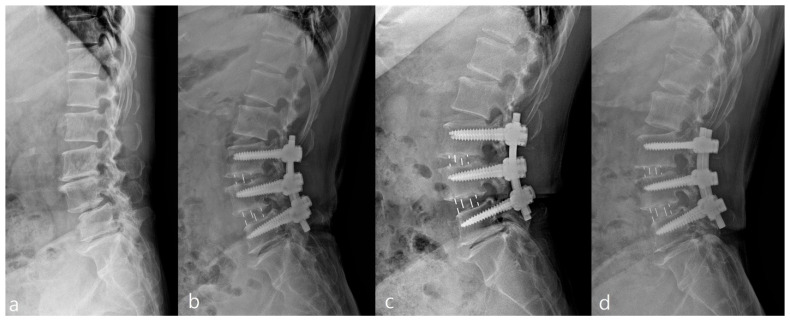
Upright lateral lumbar spine radiographs of a representative patient who underwent L3-5 OLIF. Images demonstrate early radiographic changes at different timepoints: (**a**) preoperative, (**b**) postoperative 1 month, (**c**) postoperative 3 months, and (**d**) postoperative 6 months.

Despite the advantages in lumbar lordosis and disc height restoration, our study highlights concerning early radiographic changes in the upper adjacent segment following OLIF surgery. Early postoperative increases in upper adjacent segmental lordosis, along with a higher incidence and degree of retrolisthesis, and reduced foraminal height, might suggest that higher OLIF cage acting as a potential biomechanical stressor effect on the upper adjacent segment. These findings align with previous studies indicating a correlation between fusion surgery and subsequent degenerative changes in adjacent segments [25,26]. The altered biomechanics resulting from fusion may lead to increased load and stress on adjacent segments, contributing to the observed radiographic deterioration (Figure 5). In addition to biomechanical factors, postoperative inflammatory responses should also be considered when comparing OLIF and PLIF. Minimally invasive procedures such as OLIF are generally associated with reduced systemic inflammatory response compared with more traditional open approaches, as demonstrated in recent studies. This reduced inflammatory burden may partly explain the favourable perioperative recovery profiles observed with OLIF, despite the radiographic deterioration noted in adjacent segments [27].

The radiographic changes observed in the upper adjacent segment following OLIF surgery raise important considerations for clinical practice. Surgeons should be mindful of the potential for early degenerative changes in the upper adjacent segment, particularly in patients undergoing OLIF procedures. Excessive increases in disc height and lumbar lordosis of the surgical segment may exacerbate biomechanical stress on the adjacent segment, predisposing it to degenerative changes. Long-term follow-up observation is warranted to elucidate the clinical implications of these radiographic changes and their impact on patient outcomes.

It is essential to acknowledge the limitations of this study, including its retrospective design and relatively short-term follow-up period. Future research with longer-term follow-up and prospective study designs is needed to further elucidate the clinical significance of radiographic changes in the upper adjacent segment following fusion surgery. Additionally, incorporating advanced imaging modalities such as magnetic resonance imaging and dynamic imaging techniques may provide deeper insights into the biomechanical factors contributing to adjacent segment degeneration.

## 5. Conclusions

In conclusion, OLIF demonstrates superiority over PLIF in restoring sagittal alignment and disc height, but it is associated with early radiographic changes in the upper adjacent segment. These results should be interpreted as early radiographic observations, as no significant differences were detected in clinical outcomes at 1 year. Long-term prospective studies are warranted to clarify the clinical significance of these findings.

## Figures and Tables

**Table 1:** Comparison of demographics between Groups O and P.

	Group O (*n* = 95)	Group P (*n* = 141)	*p*-Value
Age	65.07 ± 8.22	63.41 ± 7.68	0.133
Sex			0.614
male	26	41	
female	69	100	
Smoking			0.880
Non-smoker	43	67	
smoker	52	74	
Level			0.997
L3-4	29	46	
L4-5	55	83	
L3-4-5	11	12	
Lumbar Lordosis	50.75 ± 8.23	48.35 ± 12.78	0.502
Pelvic Incidence	53.47 ± 10.70	51.83 ± 10.64	0.216
Disc height (mm)	7.80 ± 3.16	7.91 ± 2.44	0.487
Upper Segment Radiological Parameter			
Foraminal height (mm)	19.53 ± 2.05	20.1 ± 3.01	0.194
Segmental Lordosis (°)	8.15 ± 3.18	7.75 ± 3.88	0.379
Flexibility (°)	7.64 ± 2.05	7.49 ± 3.88	0.515
Disc degeneration (Pfirrmann Gr.)	2.33 ± 0.062	2.44 ± 0.59	0.267
Facet sagitallization (°)	69.16 ± 9.04	71.19 ± 12.62	0.259
Facet degeneration (Weishaupt Gr.)	2.16 ± 0.69	2.36 ± 0.48	0.085

O, OLIF; P, PLIF; Gr., grade; Chi-square test, Mann–Whitney U test.

**Table 2:** Comparison of early postoperative radiographic changes between Groups O and P.

	3 Days	1 Month	3 Months	6 Months	1 Year
ΔLL(°)					
Group O	0.23 ± 4.11	1.23 ± 4.26	2.78 ± 4.32	3.74. ± 4.13	4.03 ± 4.38
Group P	0.84 ± 6.70	1.13 ± 4.87	1.23 ± 5.71	1.14 ± 5.06	1.63 ± 5.11
*p*-value	0.609	0.495	0.036 *	<0.000 *	0.001 *
ΔDisc height (mm)					
Group O	5.97 ± 3.14	5.84 ± 3.07	5.53 ± 3.53	5.52 ± 3.45	5.50 ± 3.39
Group P	3.07 ± 2.20	3.16 ± 2.23	2.96 ± 2.32	2.87 ± 2.33	2.71 ± 2.18
*p*-value	<0.000 *	<0.000 *	<0.000 *	<0.000 *	<0.000 *
ΔUpper seg. Retro-incidence (%)					
Group O	6.1	76.9	80	83.1	83.1
Group P	2.9	24.6	29.8	31.3	34.3
*p*-value	0.12	<0.000 *	<0.000 *	<0.000 *	<0.000 *
ΔUpper seg. Retrolisthesis (mm)					
Group O	0.03 ± 0.14	1.69 ± 1.09	1.80 ± 1.33	1.79 ± 1.28	1.78 ± 1.10
Group P	0.05 ± 0.78	0.29 ± 0.70	0.43 ± 0.75	0.52 ± 0.91	0.58 ± 0.98
*p*-value	0.305	<0.000 *	<0.000 *	<0.000 *	<0.000 *
ΔUpper seg. FH (mm)					
Group O	0.70 ± 0.78	−1.43 ± 2.12	−1.45 ± 2.82	−1.56 ± 2.07	−1.72 ± 1.81
Group P	1.3 ± 2.72	0.54 ± 2.53	−0.16 ± 2.42	−0.75 ± 2.72	−0.75 ± 2.72
*p*-value	0.155	<0.000 *	0.03 *	0.037 *	0.035 *
ΔUpper seg. Lordosis (°)					
Group O	1.8 ± 4.39	2.24 ± 3.55	2.33 ± 3.57	2.44 ± 3.65	2.58 ± 3.17
Group P	0.08 ± 3.35	0.23 ± 3.75	0.26 ± 3.17	0.34 ± 3.02	0.68 ± 2.63
*p*-value	0.001 *	<0.000 *	<0.000 *	<0.000 *	0.001 *

O, OLIF; P, PLIF; LL, lumbar lordosis; seg, segmental; Retro-, retrolisthesis; FH, foraminal height; * *p* < 0.05, Chi-square test, Mann–Whitney U test.

## Data Availability

The datasets generated during and/or analyzed during the current study are not publicly available but are available from the corresponding author on reasonable request.
